# ABO blood type is associated with renal outcomes in patients with IgA nephropathy

**DOI:** 10.18632/oncotarget.20701

**Published:** 2017-09-07

**Authors:** Meng Yang, Jingyuan Xie, Yan Ouyang, Xiaoyan Zhang, Manman Shi, Xiao Li, Zhaohui Wang, Pingyan Shen, Hong Ren, Wen Zhang, Weiming Wang, Nan Chen

**Affiliations:** ^1^ Department of Nephrology, Institute of Nephrology, Ruijin Hospital, Shanghai Jiao Tong University School of Medicine, Shanghai, China

**Keywords:** IgA nephropathy, ABO blood group, renal progression, end-stage renal disease, clinical follow-up, Immunology and Microbiology Section, Immune response, Immunity

## Abstract

ABO blood group antigens have been reported to be associated with inflammation and infections which have been largely implicated in the onset and progression of immune-mediated diseases. This study aimed to evaluate the association between ABO blood group and progression of IgA nephropathy (IgAN). We retrospectively enrolled 919 biopsy-proven IgAN patients with a minimum follow-up of 1 year and eGFR≥15ml/min/1.73m^2^ at the time of renal biopsy. Patients in non-B antigen group (type O/A) had lower baseline eGFR, higher systolic blood pressure (SBP), uric acid, lactate dehydrogenase, high-sensitive C-reactive protein and tumor necrosis factor-α compared to patients in B antigen group(type B/AB). After a median follow-up of 57.46 months, 124(13.5%) patients progressed to end-stage renal disease (ESRD) including 98(17.7%) in non-B antigen group and 26(7.1%) in B antigen group. Kaplan-Meier analysis showed the median ESRD-free survival time of patients in non-B antigen group was significantly shorter than patients in B antigen group [143.09±6.38 *vs* 159.05±4.94months, *p* < 0.001]. Furthermore, non-B antigen blood group was associated with an independently increased risk of ESRD (HR=2.21, 95%CI 1.35-3.62, *p* = 0.002) after fully adjusted by age, sex, SBP, eGFR, blood urea nitrogen, hypoalbuminemia, uric acid, triglycerides, hemoglobin, serum C3, urine protein, Oxford classification and glucocorticoid treatment. In conclusion, our study suggests that ABO blood type is a new risk factor for IgAN progression. IgAN patients with blood type O or A have an independent increased risk for renal function deterioration which might be explained by an increased level of inflammatory status.

## INTRODUCTION

IgA nephropathy (IgAN) is one of the most common types of primary glomerulonephritis (PGN) in the world, especially in Asia [1-3], since it was first described by Berger in 1968 [4]. Although IgAN was initially regarded as a benign disease, 20-50% patients developed to end-stage renal disease (ESRD) within 20 years after diagnosis [5-7]. We know it more clearly that IgAN is an autoimmune disease based on the findings of increasing levels of both glycto-deficient IgA1 and its antibody in patients’ kidney tissue and serum [8]. Dozens of risk factors of IgAN progression have been reported during the last decades [9-11], including systolic blood pressure (SBP), proteinuria, uric acid, glomerular filtration rate (GFR), hemoglobin, albumin and serum C3 level. However, it is still challenging to precisely predict outcomes of IgAN patients and the discovery of new risk factors are helpful for risk stratification.

ABO is the most important blood group system for the compatibility of transfusion and organ transplantation, since it was firstly reported by Landsteiner in 1900 [12]. Histo-blood group ABH (O) antigens are major histocompatibility antigens in human, expressing not only highly on red blood cell membranes but also widely on the surface of a variety of human cells and tissues, including epithelium, sensory neurons, platelet and vascular endothelium [13-15].The genes encoding the ABO alleles are located on chromosome 9q34.2 [16], composing of 7 exons. Genome-wide association studies (GWAS) [17-19] found that genetic variants at *ABO* locus were significantly associated with type 2 diabetes, venous thromboembolism and epithelial ovarian cancer. In addition, there have been increasing evidence suggesting that blood group antigens might serve as receptors for parasites, bacteria, and viruses [20, 21]. Associations between blood group antigens and immunoglobulin (Ig) superfamily, selectins, integrins, and cell surface proteoglycans have been reported over the past decades [22-24]. All these studies suggested that blood group antigens played an important role in infections and host inflammatory status. In addition, inflammation and infections have been largely implicated in the onset and progression of immune-mediated diseases. Several mechanism have been uncovered how infectious agents including viruses, bacteria, fungi and parasites trigger immune-mediated diseases [25-27]. We also know that chronic mucosal infections are important factors associated to susceptibility, severity and progression of chronic kidney disease (CKD) and IgAN [28, 29]. We therefore hypothesized ABO blood group might be associated with renal outcomes of IgAN patients and carried out this study to evaluate predictive value of ABO blood group in the progression of IgAN patients.

## RESULTS

### Baseline demographic and clinical data

The clinical characteristics of patients with IgAN at the time of renal biopsy were shown in Table 1. There were 252(27.4%) patients in type A, 273(29.7%) in type B, 93(10.1%) in type AB and 301(32.8%) in type O in our study (Supplementary Table 1). Our results showed patients with type B and AB had similar baseline clinical characteristics and renal outcomes, as well as patients with type A and O (Supplementary Table S1, Supplementary Figure 1).Therefore, we merged all type B and AB patients into B antigen group (*n* = 366) and type A and O patients into non-B antigen group (*n* = 553). Compared to patients from B antigen group, patients from non-B antigen group had lower eGFR, higher systolic blood pressure (SBP), diastolic blood pressure (DBP), uric acid and urea nitrogen (Table 1). Furthermore, we found increased levels of lactate dehydrogenase (LDH) [140.5(122.5-166.25) IU/L vs.128(121.25-142.5) IU/L, *p* = 0.006] and high-sensitive C-reactive protein (hsCRP) [0.9(0.36-2.0325) mg/Lvs.0.51(0.28-1.61) mg/L, *p* = 0.046] in patients from non-B antigen group comparing to that from B antigen group (Table 1).

**Table 1:** Baseline characteristics of IgAN patients

Variables	Blood group			*P* value	
	B antigen group (*n* = 366)		non-B antigen group (*n* = 553)		
Follow-up (months)	57.75±40.49		57.27±41.15	0.86	
Age at biopsy (years)	36.39±11.92		37.09±12.5	0.40	
Gender (Male: Female)	0.74(156:210)		1.14(294:259)	0.002*	
eGFR (mL/min/1.73m^2^)	81.28±34.68		72.47±33.78	<0.001*	
CKD stage (eGFR in mL/min/1.73m^2^)					
1-2 (≥60) (%)	245 (66.9%)		329 (59.5%)	0.001*	
3-4 (<60) (%)	121 (33.1%)		224 (40.5%)		
SBP (mm Hg)	125.26±16.58		128.77±17.83	0.003*	
DBP (mm Hg)	79.53±11.71		81.81±12.52	0.005*	
Hypertension (%)	104(28.4%)		221(40%)	<0.001*	
Blood urea nitrogen (mg/dL)	14.85(6.44-54.06)		17.37(5.88-120.45)	<0.001*^#^	
Serum uric acid (mg/dL)	6.19±1.7		6.52±1.79	0.004*	
Serum albumin (g/dL)	3.6(0.9-5)		3.6(0.7-4.9)	0.86	
Hypoalbuminemia (%)	67(18.4%)		85(15.4%)	0.24	
Serum triglycerides (mg/dL)	156.78(39.86-1140.83)		158.1(40.74-1034.54)	0.85^#^	
Serum cholesterol (mg/dL)	192.58(32.1-512.76)		194.7(32.1-696.83)	0.69^#^	
Hemoglobin (g/dL)	13.07±1.86		12.82±2.05	0.06	
Anemia (%)	136(37.3%)		240(43.5%)	0.06	
WBC (10^3^/mm^3^)	7.1(3.5-19)		7.2(3.2-22.8)	0.52^#^	
N (%)	58.90±9.32		59.70±10.07	0.23	
L (%)	31.90±8.61		31.03±9.41	0.17	
Serum IgA (mg/dL)	324(70.3-706)		322.5(68-930)	0.81^#^	
Serum C3 (mg/dL)	103(42.7-204)		100(55-218)	0.09^#^	
LDH (IU/L)	128(121.25-142.5)		140.5(122.5-166.25)	0.006*^#^	
hsCRP (mg/L)	0.51(0.28-1.61)		0.9(0.36-2.0325)	0.046*^#^	
ESR (mm/h)	13(7-22)		14(7-23)	0.75	
Urine protein excretion (g/24h)	1.04(0.03-12.7)		1.19(0.02-13.91)	0.09	
Nephrotic range proteinuria (%)	42(11.5%)		66(11.9%)	0.85	
ESRD (%)	26(7.10%)		98(17.70%)	<0.001*	
ACEI or ARB treatment (%)	308(84.20%)		471(85.20%)	0.67	
Glucocorticoid treatment (%)	211(57.70%)		309(55.90%)	0.60	
**Oxford Classification [number/(%)]**					
Mesangial hypercellularity: M1	170(46.4%)	236(42.7%)			0.26
Endocapillary hypercellularity: E1	80(21.9%)	100(18.1%)			0.16
Segmental glomerulosclerosis: S1	286(78.1%)	418(75.6%)			0.37
Tubular atrophy/Interstitial fibrosis: T1/T2	89/35(24.3/9.6%)	132/78(23.9/14.1%)			0.12

Note: Data are presented as n (%) or mean±SD or median and interquartile range. eGFR: estimated glomerular filtration rate; SBP: systolic blood pressure; DBP: diastolic blood pressure; WBC: white blood cell count; Ig: immunoglobulin; C: complement; LDH: lactate dehydrogenase; ASO: Anti-Streptolysin O; TRF: transferrin;hsCRP: high-sensitive C-reactive protein; ESR: Erythrocyte Sedimentation Rate;ACEI: angiotensin converting enzyme inhibitor; ARB, angiotensin II receptor blocker;*p<0.05; #p: *p* value was calculated by t-test for normally distributed variables after LG conversion.

No significant differences were detected between the two groups regarding to Oxford classification and treatment strategies including glucocorticoid and ACEI/ARB (Table1).

### Detection of pro-inflammatory cytokines

We also measured circulating IL-6 and TNF-α levels in 212 IgAN patients, including 128 in non-B antigen group and 84 in B antigen group. Although it was not statistically significant, the TNF-α level was higher in non-B antigen group compared to B antigen group [9.0 vs. 8.1 pg/ml]. Patients from the two groups had similar serum IL-6 levels (Table 2).

**Table 2:** Detection of proinflammatory cytokines of IgAN

Variables	Blood group		*P* value
	B antigen group (*n* = 84)	non-B antigen group (*n* = 128)	
IL-6 (pg/ml)	2.8(1.9-4.6)	2.7(1.9-6.2)	0.51^#^
TNF-α (pg/ml)	8.1(6.2-10.4)	9.0(6.5-12.8)	0.09^#^

Note: IL: interleukin; TNF: tumor necrosis factor; #p: *p* value was calculated by*t*-test for normally distributed variables after LG conversion.

### Renal outcomes

**Figure 1:**
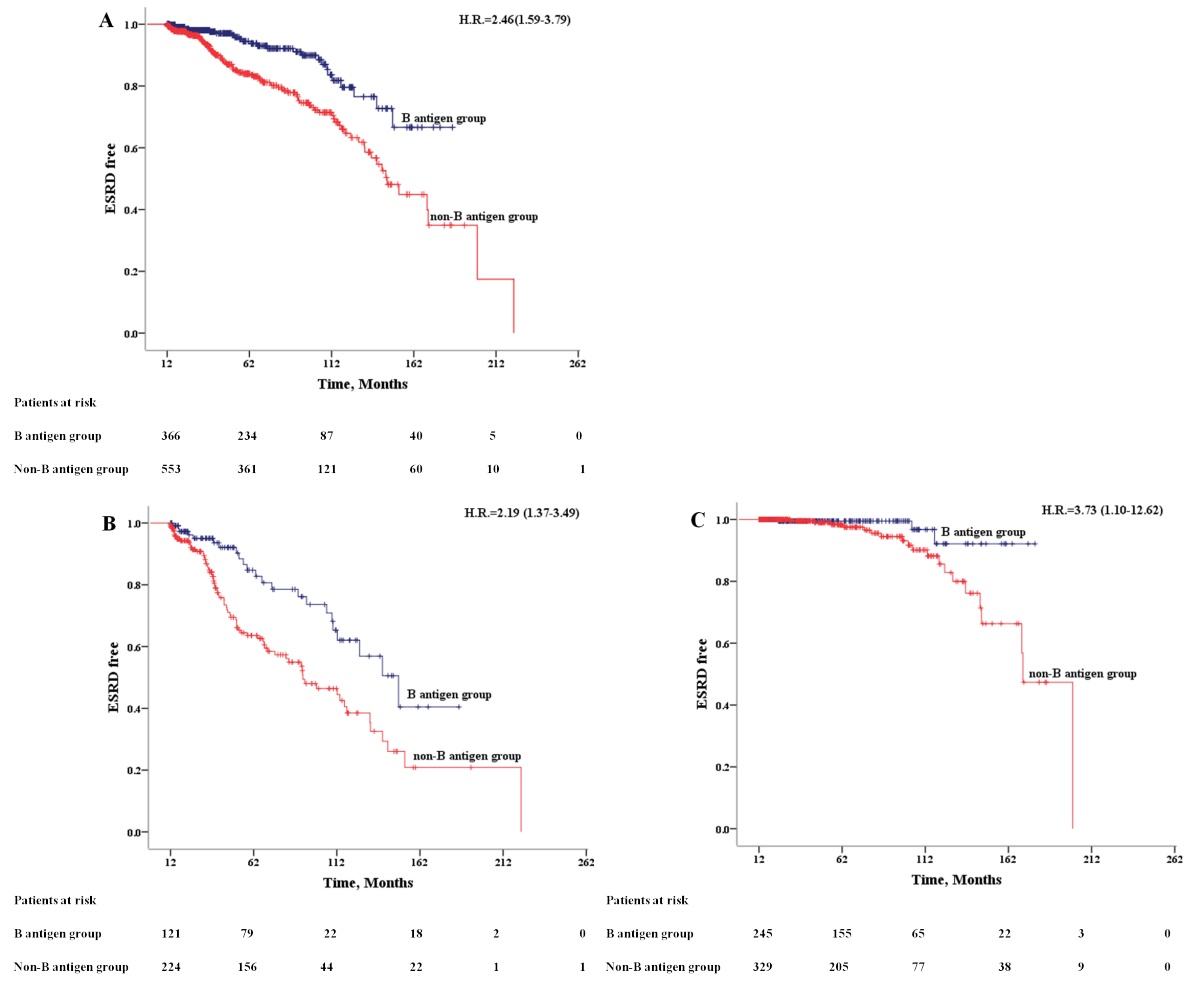
Kaplan-Meier Outcome-free Survival Curves Patients from B antigen group (dark blue); patients from non-B antigen group (red). **A**. All patients; **B**. patients in CKD 3-4 stage (eGFR < 60 ml/min/m^2^); **C**. patients in CKD 1-2 stage (eGFR >= 60 ml/min/m^2^).

After a median follow-up period of 57.46 months, totally 124 (13.5%) IgAN patients progressed to ESRD, including 42(16.7%) type A, 23(8.4%) type B, 3(3.2%) type AB and 56(18.6%) type O patients. The ESRD rate was more than two times higher in non-B antigen group (17.7%) compared to B antigen group (7.1%). Kaplan-Meier analysis showed that median ESRD-free time of patients from non-B antigen group was significantly shorter than patients from B antigen group [143.09±6.38 vs 159.05±4.94 months, *n* < 0.001] (Figure 1A). Furthermore, the association between non-B antigen group and worse renal outcomes was independent to different CKD stages (Figure 1B-1C).

By COX regression analysis, non-B antigen group were associated with an increased risk of ESRD (HR = 2.46, 95%CI 1.59-3.79) (Table 3). We took all the statistically significant variables from univariate cox analysis into multivariate cox regression proportional hazards models. The association remains robust after adjusted by age, sex and clinical indicators (SBP, eGFR, blood urea nitrogen, hypoalbuminemia, uric acid, serum triglycerides, hemoglobin, serum C3, urine protein, Oxford Classification T and glucocorticoid treatment) (HR = 2.21, 95%CI 1.35-3.62) (Table 3).

**Table 3:** Univariate and Multivariate Cox Regression analyses of baseline variables with renal outcome for ESRD.

Variable (n=919)	Univariate Cox		Multivariate Cox	
	**HR (95%CI)**	**P value**	**HR (95%CI)**	***P*-value**
Age at biopsy (years)	1.01(0.99-1.02)	0.42	0.98(0.96-0.99)	0.01*
Gender (Male)	1.67(1.17-2.41)	0.005*	1.86(1.15-3.00)	0.01*
eGFR (mL/min/1.73m^2^)	0.96(0.95-0.96)	<0.001*	0.97(0.96-0.98)	<0.001*
SBP (mm Hg)	1.02(1.01-1.03)	<0.001*	1.00(0.99-1.01)	0.83
DBP (mm Hg)	1.03(1.02-1.04)	<0.001*	--	--
Blood urea nitrogen (mg/dL)	1.07(1.06-1.08)	<0.001*	1.02(0.99-1.05)	0.14
Serum uric acid (mg/dL)	1.52(1.40-1.66)	<0.001*	1.07(0.92-1.24)	0.37
Serum albumin (g/dL)	0.61(0.49-0.76)	<0.001*	--	--
Hypoalbuminemia (%)	2.07(1.34-3.18)	0.001*	2.15(1.20-3.83)	0.01*
Serum triglycerides (mg/dL)	1.001(1-1.002)	0.02*	1.001(1-1.002)	0.07
Serum cholesterol (mg/dL)	1.001(0.998-1.004)	0.49	--	--
Hemoglobin (g/dL)	0.75(0.70-0.82)	<0.001*	0.88(0.78-0.98)	0.02*
WBC (10^3^/mm^3^)	0.93(0.86-1.02)	0.12	--	--
Serum IgA (mg/dL)	1.001(0.999-1.002)	0.48	--	
Serum C3 (mg/dL)	0.99(0.98-1.00)	0.005*	1(0.99-1.01)	0.92
Urine protein excretion (g/24h)	1.16(1.09-1.23)	<0.001*	--	--
Nephrotic range proteinuria (%)	2.40(1.55-3.74)	<0.001*	0.77(0.44-1.37)	0.38
Blood group (nonB-antigen)	2.46(1.59-3.79)	<0.001*	2.21(1.35-3.62)	0.002*
Oxford Classification: M1	0.74(0.52-1.06)	0.11	--	--
Oxford Classification: E1	0.95(0.57-1.59)	0.85	--	--
Oxford Classification: S1	1.31(0.86-1.99)	0.21	--	--
Oxford Classification: T(1 increase)	2.64(2.12-3.29)	<0.001*	2.01(1.49-2.70)	<0.001*
Oxford Classification: T1	2.12(1.36-3.32)	0.001*	--	--
Oxford Classification: T2	7.30(4.75-11.21)	<0.001*	--	--
ACEI or ARB treatment	1.08(0.63-1.86)	0.78	--	--
Glucocorticoid treatment	0.44(0.30-0.64)	<0.001*	0.61(0.37-0.995)	0.048*

Note: Data are reported as hazard ratios (HRs) and 95% confidence intervals (CIs) ;*p<0.05.

## DISCUSSION

IgAN is the most common glomerulonephritis worldwide [3]. It is also the leading cause of ESRD in China [7]. The clinical presentations and renal outcomes of IgAN are highly variable. Although dozens of risk factors have been reported, among which proteinuria, GFR and blood pressure were the most validated ones, it is still unsatisfied for clinicians to precise identify IgAN patients with high risk of progression and need be intensively treated at the time of renal biopsy. Therefore, identification of new risk factors of IgAN is crucial to improve this situation. Previous studies suggested that ABO blood antigens played an important role in infections and host inflammatory status which has been largely implicated in the onset and progression of immune-mediated diseases. In this study, we evaluated the predictive value of ABO blood group to renal outcomes based on an extended Chinese IgAN cohort with more than twenty baseline clinical parameters and long follow-up period. By COX regression analysis, we identify ABO blood type is a new risk factor for progression of IgAN. Patients with blood type O or A had a higher risk for renal function deterioration than patients with blood type B or AB, and the association is independent to age, sex, SBP, baseline eGFR, blood urea nitrogen, hypoalbuminemia, uric acid, serum triglycerides, hemoglobin, serum C3, urine protein, Oxford Classification and glucocorticoid treatment.

Infections and inflammatory status have been largely implicated in the onset and progression of immune-mediated diseases. Several mechanism have been uncovered how infectious agents including viruses, bacteria, fungi and parasites trigger immune-mediated diseases [25-27]. It has been reported that the infection, immune system and inflammatory response played a pivotal role in the pathogenesis, clinical manifestations and outcomes of IgAN [8, 30, 31]. The evidences became even stronger after the release of GWAS studies on IgAN which linked intestinal mucosal inflammatory disorders to onset of IgAN [32].

ABO blood group was firstly found to be associated with the mortality of CKD patients in a Canadian study based on 8,432 ESRD patients in 1989 [33]. In this study, the authors reported that patients with blood type AB had a clear but not statistically significant decreased risk of death. The authors explained this finding partly on that patients with blood type AB had higher likelihood of receiving a transplant. Besides, individuals with blood group A were reported to have a higher risk for *H. pylori* infection, chronic atrophic gastritis, gastric cancer and suffered more refractory iron deficiency anemia than individuals with other blood groups [34, 35]. Furthermore, individuals with blood group O was found to had higher risk to peptic ulceration due to an increasing density of colonization of epithelial cells and higher inflammatory responses (release of IL-6, IL-10 and TNF-α) to *H. pylori.* [21]. The mechanisms for the association between ABO blood type and inflammatory responses to infection have been partly uncovered. A and B antigens can be recognized as receptors for pathogens, affecting host-pathogen interactions and diseases susceptibility among individuals with different glycosylation profiles [36]. Genetic studies revealed ABO blood type was a major genetic determinant of circulation glycoprotein levels which were important in endothelial function and inflammation. These glycoproteins includs soluble intercellular adhesion molecule-1 (sICAM-1), selectins, von Willebrand factor (vWF), thrombomodulin and TNF-α [37-42]. Previous studies found increasing levels of IL-6, TNF-α and hsCRP were common in CKD patients and hsCRP was associated with progression of IgAN [43-45]. Based on these evidences, we hypothesized that it was possible that ABO blood group influenced the prognosis of IgAN via affecting inflammatory status of these patients. In our study, LDH and hsCRP were significantly elevated in non-B antigen group indicated increase of the inflammatory status. Elevation of serum LDH was regarded as an indicator of cell injury and inflammation [46]. Furthermore, although it was not significant, we found TNF-α level was also elevated in non-B antigen group compared to B antigen group. Based on these data, we assumed that ABO blood group might influence IgAN progression by its potential roles on inflammation.

In addition to ABO blood type, we also found younger male IgAN patients with lower eGFR, serum albumin, hemoglobin, and more severe tubular atrophy/interstitial fibrosis held a higher risk to progress to ESRD in IgAN patients. Interestingly, urine protein excretion did not contribute to the risk of progression in our multivariate models. The reason for this might due to its strong correlation with serum albumin and most of its variance in outcome was captured by serum albumin.

There were several limitations of our study. Firstly, the baseline characteristics between patients in B antigen group and non-B antigen group were not perfect balance. The imbalance baseline status could be partly explained by its retrospective study design. To resolve this issue we used a fully adjusted COX regression model and performed subgroup analysis by stratified patients by CKD stages. Secondly, part of patients recruited in this study didn’t have chances to measure their serum inflammatory factors because serum samples of these patients were not available.

## CONCLUSION

In conclusion, our study firstly identified that ABO blood group is a new risk factor for IgAN progression which may be explained by influencing patient’s inflammatory status. Compare to patients with blood type B/AB, patients with blood type O/A have an increased risk for disease progression of IgAN. Since ABO blood group is stable through lifetime and is one of the most readily available laboratory tests for most patients. It may represent an ideal marker for clinicians to predict disease progression. The prognostic value of ABO blood group must be further validated and its biological explanation should be elucidated.

## MATERIALS AND METHODS

### Participants

All IgAN patients recruited in this study were diagnosed and followed up in Shanghai Ruijin Hospital, Shanghai Jiao Tong University School of Medicine. The inclusion criteria were as follow: 1) IgAN was defined by dominant and at least 2+ (on a scale from 0 to 3+) mesangial staining for IgA by immunofluorescence in combination with compatible findings on light microscopy [47]; 2) Age between 10 to 75 years old; 3) eGFR≥15ml/min/1.73m^2^ at the time of biopsy; 4) Minimum follow-up of 12 months; 5) Signed informed consent. Exclusion criteria included: 1) A secondary cause of IgA was suspected, especially patients with systemic diseases; 2) Clinical and follow-up data was incomplete; 3) Patients received any immune-suppression treatment before renal biopsy. This study was in accordance with the principle of the Helsinki Declaration II. The study protocol was approved by the Institutional Review Board of Ruijin Hospital, Shanghai Jiao Tong University School of Medicine and written informed consent was obtained from each participant.

### Clinical characteristics

Baseline demographic, clinical and laboratory data were collected from all patients at the time of renal biopsy. ABO histo-blood group was determined by using standard erythrocyte antiserum agglutination methods. Histological changes were evaluatedand semi-quantitative scored according to the Oxford scoring system by experienced pathologists [48].

Estimated glomerular filtration rate (eGFR) was evaluated by The Chronic Kidney Disease Epidemiology Collaboration (CKD-EPI) Equation [49]: eGFR [ml/min/1.73m^2^] = 141 × min(Scr/κ, 1)^α^ × max(Scr/κ, 1)^-1.209^ × 0.993^Age^ × 1.018 [if female] _ 1.159 [if black], where Scr is serum creatinine (mg/dL), κ is 0.7 for females and 0.9 for males, α is -0.329 for females and -0.411 for males, min indicates the minimum of Scr/κ or 1, and max indicates the maximum of Scr/κ or 1. Chronic kidney disease (CKD) was classified based to the Kidney Disease Outcomes Quality Initiative (K/DOQI) practice guidelines [50]. Hypertension was defined as SBP≥140 mmHg or DBP≥90 mmHg (at least 2 times in different environments) or having a history of antihypertensive medication. Hypoalbuminemia was defined by serum albumin<3 g/dL. Anemia was defined by gender-specific criteria of hemoglobin concentrations<13.5 g/dL in males or<12 g/dL in females. Nephrotic range proteinuria was defined by proteinuria ≥ 3.5 g/24h. IgAN patients with hypertension (SBP≥140 mmHg or DBP≥90 mmHg) and/or proteinuria higher than 0.5g/day were treated with ACE inhibitors (ACEI) and/or angiotensin receptor blockers (ARB). Glucocortoid therapy was used if patients who did not respond to an ACEI or ARB therapy (urinary protein excretion of >1 g/day continued after 3 to 6 month therapy). Combination of immunosuppressive agents and glucocorticoids were used if patients had rapid progressing glomerulonephritis.

### Study end points

The study endpoint was ESRD defined as eGFR<15ml/min/1.73m^2^ or need renal replacement therapy (dialysis or renal transplantation). Patients were censored at the time of death or ESRD or loss of follow-up.

### Detection of circulating interleukin-6 (IL-6) and tumor necrosis factor-α (TNF-α) levels

5ml venous bloodwas collected from IgAN patients on the morning of renal biopsy. Plasma was extracted and divided into aliquots. The serum samples were stored at -80°C fridge until they were tested. IL-6 and TNF-αlevels were measured by a sequential solid phase chemiluminescent assay performed by IMMULITE 1000 Analyzer (Siemens Medical Solutions Diagnostics) using corresponding kits by professionals according to the manufacturer’s instructions. The kits were purchased from SIEMENS (IMMULITE 1000 IL-6/ TNF-α).

### Statistical analyses

**Figure 2:**
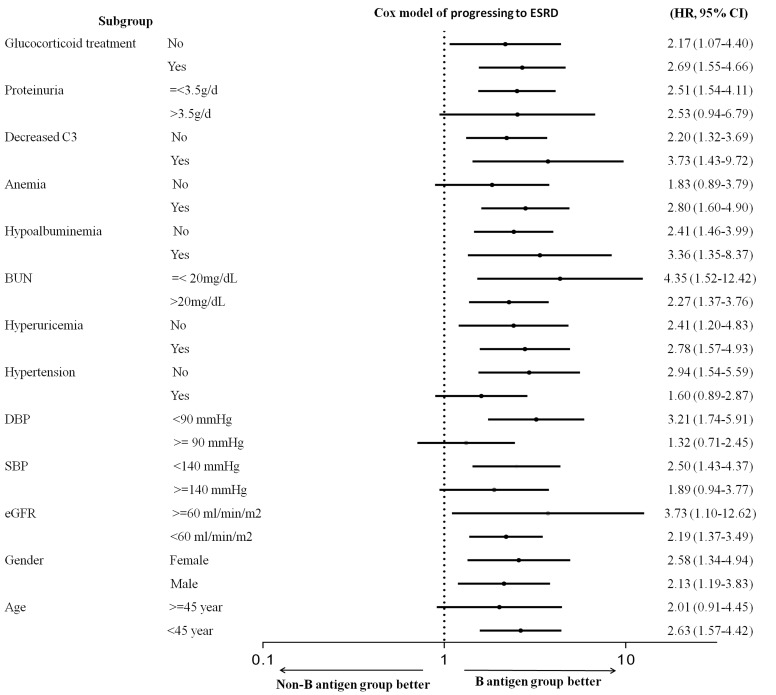
Stratified Cox model of progressing to ESRD (non-B antigen group vs B antigen group) Log-scale plot of hazard ratios (HRs) and 95% CIs for disease progression.

Statistical analysis was performed using SPSS version 17.0 (SPSS Inc., Chicago, Illinois, USA). The distributions of quantitative variables were assessed for normality. Continuous data were expressed as mean ± standard deviation (SD) (normally distributed variables) or median and interquartile range (non-normally distributed variables). Student t-test (normally distributed variables) or Mann–Whitney test (non-normally distributed variables) were used when compared with two groups. Categorical data were expressed as frequencies and percentages (%) and compared by using a standard chi-squared test. Probabilities of cumulative renal survival curves were generated by the Kaplan-Meier method, and log-rank test was used when comparing survival time between two groups (Figure 2). Univariate and multivariate Cox regression proportional hazards models were builtto evaluate independent risk factors of ESRD. The results of Cox regressionanalyses were expressed as hazard ratios (HRs) with 95% confidence intervals (CIs). In all analyses, a two sided *P* < 0.05 was considered as statistically significant.

## SUPPLEMENTARY MATERIALS FIGURE AND TABLE




