# Supplementary material for: ABO blood type is associated with renal outcomes in patients with IgA nephropathy
Source: Oncotarget. 2017 Sep 7;8(43):73603–12. doi: 10.18632/oncotarget.20701 (PMC5650285; doi:10.18632/oncotarget.20701)
Supplement: Supplementary file 1 [file oncotarget-08-73603-s001.pdf]

ABO blood type is associated with renal outcomes in patients with IgA nephropathy

SUPPLEMENTARY MATERIALS

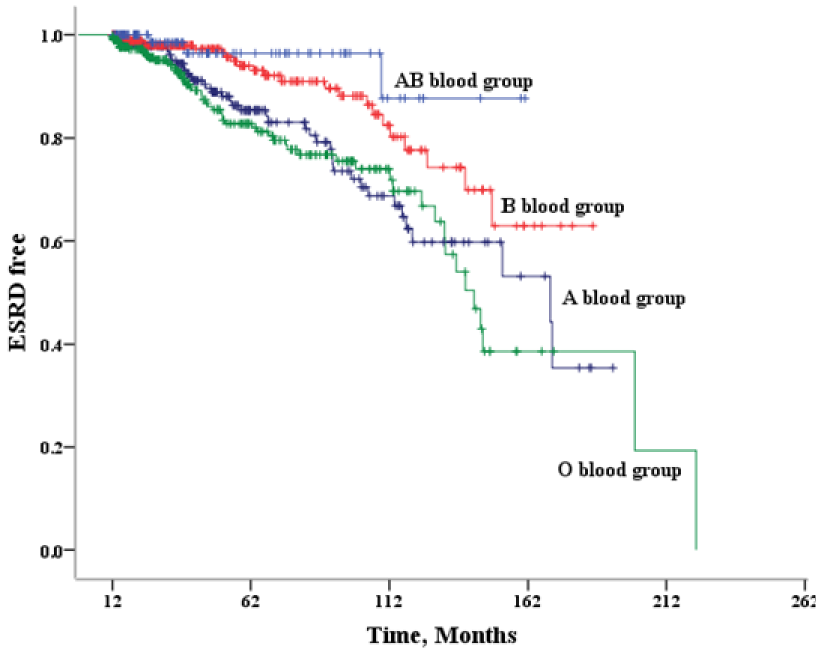

Supplementary Figure 1: Kaplan-Meier Outcome-free Survival Curves. Patients from blood group AB (blue); patients from blood group B (red); Patients from blood group A (dark blue); Patients from blood group O (green).

For Supplementary Tables see in Supplementary Files
