# Supplementary material for: ABO blood type is associated with renal outcomes in patients with IgA nephropathy
Source: Oncotarget. 2017 Sep 7;8(43):73603–12. doi: 10.18632/oncotarget.20701 (PMC5650285; doi:10.18632/oncotarget.20701)
Supplement: Supplementary file 2 [file oncotarget-08-73603-s002.docx]

**Supplementary Table 1**: Baseline characteristics of IgAN patients

| **Variables** | **Blood group** | | | | | | | | | | | | | | | | | | |
| --- | --- | --- | --- | --- | --- | --- | --- | --- | --- | --- | --- | --- | --- | --- | --- | --- | --- | --- | --- |
|  | **B antigen group(n=366)** | | | | | | | | | |  | | | | **non-B antigen group(n=553)** | | | | |
|  | **AB (n=93)** | | | | | | **B (n=273)** | **P* value** | | |  | | | | **A (n=252)** | | | **O (n=301)** | **P# value** |
| Follow-up (months) | 52.43±37.92 | | | | | | 59.57±41.24 | 0.14 | | |  | | | | 58.06±41.78 | | | 56.6±40.67 | 0.68 |
| Age at biopsy (years) | | 35.3±11.97 | | | | | 36.76±11.9 | 0.31 | | |  | | | | 37.08±13.37 | | | 37.1±11.74 | 0.99 |
| Gender (Male: Female) | | | 0.58(34:59) | | | | 0.81(122:151) | 0.17 | | |  | | | | 1.25(140:112) | | | 1.05(154:147) | 0.30 |
| Body mass index (kg/m2) | | | 22.72±3.47 | | | | 22.73±3.22 | 0.99 | | |  | | | | 23.24±3.41 | | | 23.26±3.43 | 0.95 |
| Serum creatinine (mg/dL) | | | 0.96(0.49-4.06) | | | | 1.05(0.44-4.24) | 0.65 | | |  | | | | 1.13(0.49-4.12) | | | 1.2(0.35-5.18) | 0.37 |
| eGFR (mL/min/1.73m^2^) | | | | 83.55±37.69 | | | 80.51±33.63 | 0.49 | | |  | | | | 73.67±33.24 | | | 71.46±34.25 | 0.44 |
| CKD stage (eGFR in mL/min/1.73m^2^) | | | | | |  |  |  | | |  | | | |  | | |  |  |
| 1-2 (≥60) (%) | 58 (62.4%) | | | | | | 187 (68.5%) | 0.06 | | |  | | | | 154 (61.1%) | | | 175 (58.1%) | 0.27 |
| 3-4 (<60) (%) | 35 (37.6%) | | | | | | 86 (31.5%) |  |  |  |  | | | | 98 (38.9%) | | | 126 (41.9%) |  |
| SBP (mm Hg) | 125.04±16.77 | | | | | | 125.33±16.55 | 0.88 | | |  | | | | 129.5±18.68 | | | 128.16±17.09 | 0.38 |
| DBP (mm Hg) | 78.16±12.57 | | | | | | 80±11.39 | 0.19 | | |  | | | | 81.41±12.48 | | | 82.15±12.57 | 0.49 |
| MAP (mm Hg) | 93.79±13.32 | | | | | | 95.11±12.26 | 0.38 | | |  | | | | 97.44±13.55 | | | 97.48±13.19 | 0.97 |
| Hypertension (%) | 22/93(23.7%) | | | | | | 82/273(30%) | 0.24 | | |  | | | | 100/252(39.7%) | | | 121/301(40.2%) | 0.90 |
| Blood urea nitrogen (mg/dL) | | | | 14.29(7.28-53.5) | | | 15.13(6.44-54.06) | 0.32 | | |  | | | 16.95(5.88-120.45) | | | | 17.65(5.88-76.47) | 0.14 |
| Serum uric acid (mg/dL) | | | 6.09±1.61 | | | | 6.22±1.73 | 0.51 | | |  | | | | 6.58±1.86 | | | 6.48±1.73 | 0.49 |
| Hyperuricemia (%) | 30/93(32.3%) | | | | | | 102/272(37.5%) | 0.36 | | |  | | | | 98/252(38.9%) | | | 123/300(41.0%) | 0.61 |
| Serum total protein (g/dL) | | | 6.19±0.88 | | | | 6.28±0.88 | 0.42 | | |  | | | | 6.29±0.91 | | | 6.26±0.89 | 0.66 |
| Serum albumin (g/dL) | | 3.5(1.8-5) | | | | | 3.6(0.9-5) | 0.12 | | |  | | | | 3.6(0.7-4.9) | | | 3.6(1-4.9) | 0.72 |
| Hypoalbuminemia (%) | | 19/93(20.4%) | | | | | 48/272(17.6%) | 0.55 | | |  | | | | 41/252(16.3%) | | | 44/300(14.7%) | 0.60 |
| Serum triglycerides (mg/dL) | | | | 147.03(39.86-654.56) | | | 159.43(42.52-1140.83) | | | 0.19 |  | 176.26(40.74-752.88) | | | | 147.48(50.49-1034.54) | | | 0.05* |
| Serum cholesterol (mg/dL) | | | | 190.64(100.15-474.48) | | | 193.74(32.1-512.76) | | 0.75 | |  | | 196.25(59.55-576.57) | | | | 192.38(32.1-696.83) | | 0.67 |
| Hemoglobin (g/dl) | 12.83±1.71 | | | | | | 13.15±1.91 | 0.15 | | |  | | | | 12.84±1.98 | | | 12.8±2.11 | 0.83 |
| Anemia (%) | 38/93(40.9%) | | | | | | 98/272(36.0%) | 0.41 | | |  | | | | 110/252(43.7%) | | | 130/300(43.3%) | 0.94 |
| Plt (10^3^/mm3) | 214.86±60.73 | | | | | | 218.92±63.24 | 0.59 | | |  | | | | 216.73±61.55 | | | 215.29±64.58 | 0.79 |
| WBC (10^3^/mm3) | 7.11(4.3-19) | | | | | | 7.1(3.5-17.7) | 0.54 | | |  | | | | 7.3(3.7-20.1) | | | 7.1(3.2-22.8) | 0.78 |
| Serum IgA (mg/dL) | 317.5(70.3-688) | | | | | | 325(112-706) | 0.56 | | |  | | | | 320.5(99-930) | | | 322.5(68-781) | 0.81 |
| Serum IgM (mg/dL) | | 118(30-400) | | | | | 126.5(25-714) | 0.90 | | |  | | | | 116.5(29-408) | | | 126(14-479) | 0.17 |
| Serum IgG (mg/dL) | 1123.95±445.46 | | | | | | 1105.83±358.08 | 0.70 | | |  | | | | 1110.19±331.67 | | | 1128.1±346.69 | 0.55 |
| Serum C3 (mg/dL) | 102(61.9-198) | | | | | | 104(42.7-204) | 0.64 | | |  | | | | 101(62-218) | | | 100(55-181) | 0.29 |
| Serum C4 (mg/dL) | 22.1(12-56) | | | | | | 24.45(10.25-63) | 0.11 | | |  | | | | 23.4(10.6-75.7) | | | 23.7(8-80) | 0.58 |
| Urine protein excretion (g/24h) | | | | | 1.08(0.03-8.53) | | 1.02(0.04-12.7) | 0.69 | | |  | | | | 1.18(0.02-10.1) | | | 1.2(0.05-13.91) | 0.32 |
| ESRD(%) | 3/93(3.2%) | | | | | | 23/273(8.4%) | 0.09 | | |  | | | | 42/252(16.7%) | | | 56/301(18.6%) | 0.55 |
| ACEI or ARB treatment (%) | | | | 73/93(78.5%) | | | 235/273(86.1%) | 0.08 | | |  | | | | 213/252(84.5%) | | | 258/301(85.7%) | 0.70 |
| Glucocorticoid treatment (%) | | | | 61/93(65.6%) | | | 150/273(54.9%) | 0.07 | | |  | | | | 143/252(56.7%) | | | 166/301(55.1%) | 0.71 |
| Data are presented as n (%) or mean±SD or median and interquartile range. eGFR: estimated glomerular filtration rate; SBP: systolic blood pressure; DBP: diastolic blood pressure; PLT: platelet count; WBC: white blood cell count; Ig: immunoglobulin; C: complement; LDH: lactate dehydrogenase; hsCRP: high-sensitive C-reactive protein; ESR: Erythrocyte Sedimentation Rate; ACEI: angiotensin converting enzyme inhibitor; ARB, angiotensin II receptor blocker;*: p<0.05; P* value: p value from B vs AB blood group; P# value: p value from A vs O blood group | | | | | | | | | | | | | | | | | | | |

**Supplementary Table 2**. Histological changes (Oxford classification).

| **Variables(n=919)** | **Blood group** | | | | **P** |
| --- | --- | --- | --- | --- | --- |
|  | **A (n=252)** | **B(n=273)** | **AB (n=93)** | **O (n=301)** | **value** |
| **Oxford Classification [number/(%)]** |  |  |  |  |  |
| Mesangialhypercellularity: M1 | 105(41.7%) | 124(45.4%) | 46(49.5%) | 131(43.5%) | 0.59 |
| Endocapillaryhypercellularity: E1 | 46(18.3%) | 55(20.1%) | 25(26.9%) | 54(17.9%） | 0.26 |
| Segmental glomerulosclerosis: S1 | 190(75.4%) | 213(78%) | 73(78.5%) | 228(75.7%) | 0.85 |
| Tubular atrophy/Interstitial fibrosis: T1/T2 | 61/31(24.2/12.3%) | 65/26(23.8/9.5%) | 24/9(25.8/9.7%) | 71/47(23.6/15.6%) | 0.44 |

**Supplementary Table 3.** Detection of pro-inflammatory cytokines of IgAN patients.

| **Variables** | **Blood group** | | | | | **P  value** |
| --- | --- | --- | --- | --- | --- | --- |
|  | **B antigen group** | |  | **non-B antigen group** | |  |
|  | **AB (n=20)** | **B(n=64)** |  | **A(n=56)** | **O (n=72)** |  |
| IL-6 (pg/ml) | 2.9(1.9-3.5) | 2.6(1.9-4.6) |  | 2.6(1.9-6.3) | 2.8(1.9-5.7) | 0.92^#^ |
| TNF-α (pg/ml) | 7.8(5.6-10.2) | 8.1(6.2-10.8) |  | 9.1(6.8-12.9) | 8.9(6.3-12.8) | 0.28^#^ |
| Note: IL: interleukin; TNF: Tumor Necrosis Factor; ^#^p: p value was calculated by t-test for normally distributed variables after LG conversion. | | | | | | |
